# Designing for calm: exploring the impact of colored light on autonomic regulation and anxiety in patients awaiting cataract surgery

**DOI:** 10.3389/fpsyg.2026.1731763

**Published:** 2026-07-23

**Authors:** Gilles Guerrier, Eva Hardy, Philomène Robert, Nathalie Junod Ponsard, Patrick Renaud, Pierre-Raphaël Rothschild, Christophe Baillard

**Affiliations:** 1 Department of Anaesthesia and Intensive Care, Hôpital Cochin, Assistance Publique-Hôpitaux de Paris, Paris, France; 2 Ecole Nationale Supérieure des Arts Décoratifs, Paris, France; 3 Department of Ophthalmology, Hôpital Cochin, Assistance Publique-Hôpitaux de Paris, Paris, France

**Keywords:** anxiety, heart rate variability, light design, patient experience, preoperative environment

## Abstract

**Objective:**

To explore whether immersive colored light can be used as a design intervention to reduce preoperative anxiety and modulate autonomic nervous system activity in patients awaiting cataract surgery.

**Background:**

Preoperative anxiety is common and often underestimated. Environmental factors such as lighting may influence patients' emotional state and physiological stress responses. Integrating light-based strategies into preoperative spaces represents an opportunity for evidence-based design.

**Methods:**

Thirty patients awaiting cataract surgery were exposed to immersive colored light (blue, green, purple, red, orange, and yellow) for 10 min each in randomized order, with ambient light serving as the control condition. Anxiety was assessed before and after each exposure using a verbal analog scale (VAS). Autonomic regulation was evaluated using heart rate variability (HRV), measured as the root mean square of successive differences (RMSSD) with a wearable sensor. Subjective color perception and color preference were also recorded. Anxiety and autonomic outcomes were analyzed using linear mixed-effects models including lighting condition and color preference as fixed effects and participant as a random intercept.

**Results:**

Baseline anxiety and RMSSD under ambient light conditions were 5.6 ± 2.1 VAS points and 24.8 ± 9.3 ms, respectively. Linear mixed-effects modeling demonstrated a significant effect of lighting condition on both anxiety and HRV (both *p* < 0.05). Blue light exposure was associated with significantly lower anxiety scores compared with ambient light [β = −2.87 VAS points; 95% CI (−4.52 to −1.22); *p* = 0.001] and higher RMSSD values [β = +5.8 ms; 95% CI (2.1 to 9.5); *p* = 0.003]. Yellow (β = −6.9 ms; *p* < 0.001) and red light (β = −2.1 ms; *p* = 0.038) were associated with lower RMSSD values. Color preference was not independently associated with anxiety (*p* = 0.37) or RMSSD (*p* = 0.46). Anxiety scores were inversely correlated with RMSSD values (*r* = −0.42, *p* = 0.02), whereas no significant correlation was observed with heart rate (*r* = 0.18, *p* = 0.34). Blue light was predominantly perceived as calming, while red and yellow light were most frequently perceived as stimulating.

**Conclusions:**

This exploratory study suggests that immersive colored light may influence both subjective anxiety and autonomic regulation in patients awaiting cataract surgery. Blue light was associated with reduced anxiety and enhanced parasympathetic activity independently of color preference. These findings support the potential of adaptive lighting design as a non-pharmacological strategy to improve the perioperative patient experience.

## Background

Preoperative anxiety is highly prevalent, yet frequently overlooked in perioperative care, affecting between 10% and 80% of surgical patients depending on the procedure and assessment method. Expectations, information quality, and uncertainty are recognized determinants of perioperative emotional distress [Krupić and Krupić (2025); Snyder-Ramos et al., 2005].

Traditionally managed through pharmacological means, anxiety reduction strategies rarely incorporate environmental design factors (Journal of Clinical Medicine, 2024; Ulrich et al., 2008). Research in environmental psychology and neuroscience indicates that light exposure can modulate circadian rhythms, emotional states, and autonomic nervous system activity (Chalmers et al., 2014; Schäfer and Kratky, 2006). Short-wavelength (blue) light, in particular, interacts with intrinsically photosensitive retinal ganglion cells and neural pathways that regulate alertness and stress responses (Petrowski et al., 2023; Edelhäuser et al., 2013; Cajochen et al., 2005). From a design perspective, lighting is a modifiable environmental parameter that may support patient comfort, safety, and overall satisfaction in healthcare settings. Immersive colored light offers a low-cost, non-invasive intervention with potential to enhance the healing environment. Although colored light exposure has previously been investigated in environmental psychology and autonomic regulation research, very few studies have evaluated immersive colored lighting as a perioperative environmental intervention. In particular, the acute effects of colored light on both subjective anxiety and heart rate variability in patients immediately awaiting surgery remain insufficiently explored. Reduced HRV has consistently been associated with anxiety, impaired emotional regulation, and increased physiological stress, supporting its use as a surrogate marker of autonomic adaptation (Beauchaine and Thayer, 2015). This study aimed to evaluate the feasibility and physiological impact of colored light immersion inpatients awaiting cataract surgery.

## Methods

**Figure 1:**
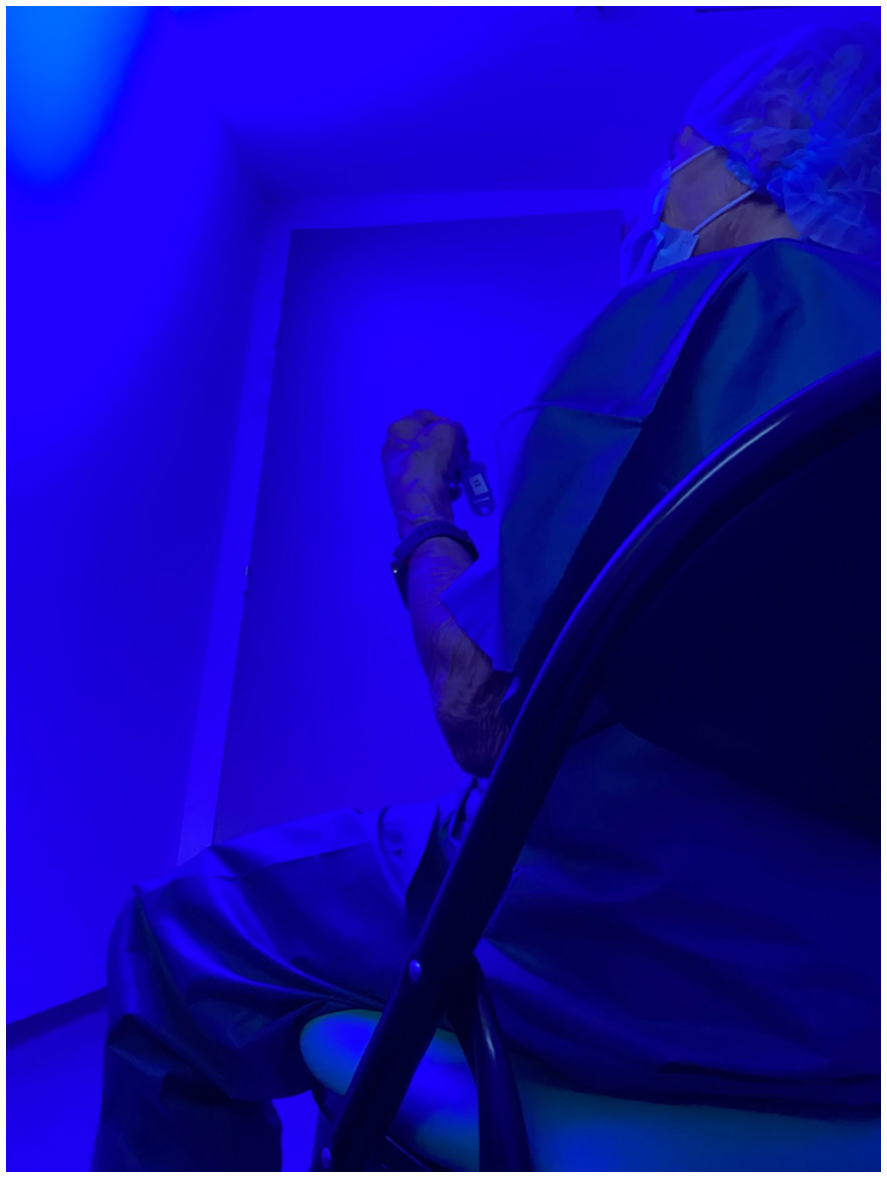
Patient during the blue light immersion.

This pilot study was approved by the institutional ethics committee (ref CPP Ouest 6 – 1214 MS2 – December 21, 2021). Written informed consent was obtained. Eligible participants were adults scheduled for elective cataract surgery. Exclusion criteria included beta-blocker treatment or pacemaker. Cataract surgery patients were specifically selected because they remain conscious throughout the perioperative pathway and frequently experience anticipatory anxiety despite the minimally invasive nature of the procedure. Participants were exposed to six colors (blue, green, purple, red, orange, yellow) using LED projectors (MINIKOLOR Starway^®^) controlled via computer, in randomized sequence, each for 10 min. Ambient light served as control. In this study, immersive colored light referred to full-room ambient illumination generated by ceiling-directed LED projectors producing a uniform colored environment around the seated participant. The intervention did not involve wearable devices, goggles, or focal visual stimulation. Participants sat in a comfortable chair under uniform ceiling-directed illumination (see Figure 1). Each lighting condition was administered for 10 minutes. This duration was selected to remain compatible with routine preoperative workflows while providing sufficient time for participants to perceive the environmental change and for potential autonomic responses to emerge. Subjective anxiety was evaluated using a Verbal Analog Scale (VAS) administered immediately before and after each light immersion. VAS is a validated and widely used instrument for the measurement of subjective anxiety (Facco et al., 2011).

Autonomic regulation was assessed through heart rate variability, specifically the root mean square of successive differences (RMSSD), measured with a wearable GARMIN^®^ device. RMSSD is a time-domain HRV metric reflecting short-term parasympathetic modulation and vagally mediated autonomic regulation. Reduced HRV has been associated with anxiety, stress, and impaired emotional regulation. Heart rate values were also extracted from the same device. In addition, subjective perception of each lighting condition was assessed immediately after exposure using a brief questionnaire. Participants were asked to classify their emotional response to each color into one of three predefined categories: calming, neutral, or stimulating. These categories were selected to capture the overall emotional valence of the lighting condition while maintaining a simple and clinically interpretable assessment. The frequency of responses within each category was recorded and summarized descriptively for each lighting condition. Emotional classifications (“calming”, “neutral”, or “stimulating”) were analyzed using a multinomial mixed-effects model with participant included as a random intercept to account for repeated observations within individuals. Lighting condition was entered as the fixed effect, and overall significance was assessed using likelihood ratio tests. Color preference scores were incorporated as fixed effects in linear mixed-effects models. Although individual preferences contributed modestly to subjective responses, the effects of lighting condition on anxiety and HRV remained significant after adjustment, suggesting that the observed physiological responses were not solely attributable to color preference. For anxiety outcomes, the following model was used:


$$\begin{array}{l} \text{VASij}=\text{β}0+\beta 1\left(\text{Colorij}\right)+\beta 2\left(\text{Preferenceij}\right)+ui+\varepsilon ij \end{array}$$

where ui represents the participant-specific random effect and εij the residual error.

A similar model was applied to RMSSD and heart rate outcomes.

This analytical framework appropriately accounts for the repeated-measures structure of the study and allows direct estimation of color-specific effects while adjusting for individual color preference. Effect estimates are reported with 95% confidence intervals. Statistical significance was defined as *p* < 0.05.

As this was an exploratory pilot study, no formal a priori power analysis was performed.

## Results

### Participant characteristics

Thirty patients scheduled for elective cataract surgery were included in the study. The mean age was 66 ± 9 years (range 56–74 years), and 48% were male. All participants completed the entire protocol, including exposure to all six color conditions and completion of the subjective evaluation questionnaires. No adverse events or discomfort related to the lighting intervention were reported. Baseline anxiety score under ambient light conditions was 5.6 ± 2.1 on the verbal analog scale (VAS). Baseline heart rate averaged 74 ± 11 beats per minute, and baseline RMSSD was 24.8 ± 9.3 ms (Table 1).

**Table 1:** Variation of HRV parameter (RMSSD) measured during exposure to colored light compared to ambient light.

Type of light	VAS	VAS variation	*p*-value	RMSSD	RMSSD variation	*p*-value	HR	HR variation	*p*-value
Ambiant light	5 (2)	–	–	29.6 (18.1)	–	–	78 (17)	–	
Blue (*n =* 30)	2 (3)	3 (2)	0.02	35.7 (16.1)	6.1 (3.5)	0.003	74 (16)	4 (3)	0.07
Green (*n =* 30)	6 (3)	1 (2)	0.57	27.8 (21.3)	−2.2 (4.7)	0.47	80 (21)	2 (7)	0.55
Purple (*n =* 29)	5 (2)	0 (2)	0.32	31.5 (22.2)	1.4 (4.6)	0.24	69 (22)	11 (4.6)	0.24
Red (*n =* 29)	5 (3)	0 (2)	0.09	27.8 (18.2)	−2.3 (3.3)	0.04	77 (18)	1 (3)	0.19
Orange (*n =* 28)	4 (2)	1 (3)	0.77	30.6 (20.0)	0.8 (4.5)	0.86	75 (20)	3 (5)	0.48
Yellow (*n =* 27)	7 (4)	2 (4)	0.01	23.2 (24.8)	−7.4 (8.6)	< 0.001	85 (29)	7 (8)	0.06

VAS, Verbal Analog Scale; RMSSD, Root mean square of standard deviation; HR, Heart rate.

Variation after 10 minutes colored light immersion compared to ambient light.

Standard deviation are noted in parentheses.

Significance level *p* < 0.05.

Participants reported distinct emotional responses across lighting conditions. Blue light was classified as calming by 24 of 30 participants (80%), neutral by 5 participants (17%), and stimulating by 1 participant (3%). In contrast, red and yellow light were predominantly classified as stimulating, being rated as such by 20 of 30 participants (67%) and 19 of 30 participants (63%), respectively. Green, purple, and orange light elicited more heterogeneous responses and were most frequently classified as neutral, with no single emotional category predominating.

The linear mixed-effects model demonstrated a significant overall effect of lighting condition on subjective anxiety (*p* < 0.001). Blue light exposure was associated with significantly lower anxiety scores than ambient light (β = −2.87 VAS points; 95% CI [−4.52 to −1.22]; *p* = 0.001). Estimated marginal means further showed significantly lower anxiety scores under blue light compared with red (adjusted *p* < 0.001) and yellow light (adjusted *p* = 0.002). No statistically significant differences were observed between blue and green [mean difference = −1.20 VAS points; 95% CI (−2.84 to 0.44); adjusted *p* = 0.12], purple [mean difference = −1.35 VAS points; 95% CI (−3.01 to 0.31); adjusted *p* = 0.18], or orange light [mean difference = −1.55 VAS points; 95% CI (−3.22 to 0.12); adjusted *p* = 0.074] following adjustment for multiple comparisons.

### Effects of colored light on heart rate variability

Linear mixed-effects analysis demonstrated a significant overall effect of lighting condition on RMSSD values (χ^2^ = 17.3, df = 6, *p* = 0.008).

Blue light exposure was associated with significantly higher RMSSD values compared with ambient light [β = +5.8 ms; 95% CI (2.1 to 9.5); *p* = 0.003], indicating enhanced parasympathetic modulation. In contrast, yellow light was associated with significantly lower RMSSD values [β = −6.9 ms; 95% CI (−11.3 to −2.5); *p* < 0.001], while red light produced a smaller but still significant reduction [β = −2.1 ms; 95% CI (−4.0 to −0.2); *p* = 0.038].

Estimated marginal means revealed the highest RMSSD values during blue light exposure (30.9 ± 1.8 ms) and the lowest values during yellow light exposure (17.4 ± 1.9 ms). Pairwise comparisons confirmed significantly higher RMSSD values under blue light than under yellow light (adjusted *p* = 0.002) and red light (adjusted *p* = 0.03) following Bonferroni correction.

### Heart rate responses

No significant effect of lighting condition was observed on heart rate (χ^2^ = 4.1, df = 6, *p* = 0.66). Similarly, color preference was not associated with heart rate values [β = 0.07 bpm per preference unit; 95% CI (−0.18 to 0.32); *p* = 0.58].

### Association between anxiety and autonomic parameters

Exploratory Pearson correlation analysis demonstrated a moderate inverse correlation between VAS anxiety scores and RMSSD values (*r* = −0.42, *p* = 0.02), indicating that higher subjective anxiety was associated with lower parasympathetic activity. In contrast, no significant correlation was observed between anxiety scores and heart rate (*r* = 0.18, *p* = 0.34).

### Subjective perception of colors

The distribution of emotional responses differed markedly across lighting conditions. Blue light was most frequently classified as calming (24/30, 80%), followed by green (18/30, 60%) and purple (15/30, 50%). In contrast, red (20/30, 67%), yellow (20/30, 67%), and orange (17/30, 57%) were predominantly classified as stimulating.

A multinomial mixed-effects model including participant as a random intercept demonstrated a significant overall effect of lighting condition on emotional classification (likelihood ratio test: χ^2^ = 68.4, df = 10, *p* < 0.001). Compared with ambient light, blue light showed the highest probability of being classified as calming rather than stimulating (OR = 8.1, 95% CI 3.4–19.2, *p* < 0.001). Green light also significantly increased the probability of a calming classification (OR = 3.6, 95% CI 1.6–8.0, *p* = 0.002), whereas purple showed an intermediate profile that did not significantly differ from green after adjustment (OR = 1.8, 95% CI 0.9–3.7, *p* = 0.11). Conversely, red (OR = 0.18, 95% CI 0.08–0.42, *p* < 0.001), yellow (OR = 0.21, 95% CI 0.09–0.48, *p* < 0.001), and orange (OR = 0.39, 95% CI 0.18–0.84, *p* = 0.016) were significantly less likely to be classified as calming and were predominantly perceived as stimulating.

### Feasibility and acceptability

The intervention was well tolerated by all participants. No participant requested interruption of the protocol, and all lighting conditions were completed successfully. Overall acceptability was high, supporting the feasibility of implementing immersive colored lighting interventions within routine preoperative care pathways.

## Discussion

The present findings are broadly consistent with previous studies suggesting that environmental lighting may influence autonomic regulation and emotional processing (Beauchaine and Thayer, 2015). Experimental studies conducted in healthy volunteers have reported alterations in heart rate variability under different colored lighting conditions, although results remain heterogeneous due to differences in light intensity, exposure duration, participant characteristics, and methodological approaches. Our findings extend this literature by investigating patients in a real-world perioperative setting, a context characterized by anticipatory stress and increased autonomic activation. Unlike laboratory-based experiments, the present study evaluated individuals facing an imminent surgical procedure, thereby providing insights into the potential relevance of lighting interventions in clinical practice.

The observed increase in RMSSD during blue light exposure is particularly noteworthy because reduced HRV has consistently been associated with anxiety disorders, impaired emotional regulation, and heightened physiological stress responses. Consequently, the increase in parasympathetic activity observed under blue light may reflect a shift toward a more adaptive autonomic state. The increase in RMSSD observed during blue light exposure may indicate enhanced parasympathetic modulation. This interpretation is consistent with previous evidence linking higher HRV with improved emotional regulation and lower anxiety levels. The perioperative environment has traditionally been designed around technical and logistical constraints, whereas psychological and emotional dimensions have often received less attention. Nevertheless, increasing evidence suggests that environmental factors—including architecture, acoustics, visual design, natural light, and color—can significantly influence patient experience and physiological responses.

An additional finding of the present study was the relationship between subjective anxiety and autonomic regulation. A moderate inverse correlation between VAS anxiety scores and RMSSD values indicates that participants reporting higher anxiety levels tended to exhibit lower parasympathetic activity. This observation is consistent with previous studies linking reduced heart rate variability to anxiety, stress, and impaired emotional regulation (Beauchaine and Thayer, 2015). In contrast, no significant correlation was observed between anxiety scores and heart rate, suggesting that HRV may be more sensitive than heart rate alone for detecting emotional and autonomic changes in the perioperative setting. The concordance between lower anxiety scores and higher RMSSD values observed during blue light exposure further supports the physiological relevance of HRV as an objective marker of emotional state and strengthens the plausibility of a link between environmental lighting and autonomic regulation.

Preoperative waiting areas represent a particularly important target for environmental interventions. During this period, patients frequently experience uncertainty, fear, and anticipatory anxiety while receiving relatively little active clinical care. Environmental modifications capable of reducing stress without pharmacological intervention may therefore offer substantial benefits. From this perspective, immersive colored lighting represents an attractive intervention because it is non-invasive, inexpensive, easily adaptable, and potentially scalable across healthcare facilities. Unlike pharmacological anxiolysis, environmental interventions do not expose patients to medication-related adverse effects and may be integrated seamlessly into existing perioperative pathways.

Several mechanisms may explain the observed association between blue light exposure and reduced anxiety. First, short-wavelength light stimulates intrinsically photosensitive retinal ganglion cells containing melanopsin (Cajochen et al., 2005). These specialized photoreceptors project to multiple brain regions involved in circadian regulation, emotional processing, attention, and autonomic control. These include hypothalamic and preoptic regions involved in homeostatic regulation, the reticular activating system, autonomic nuclei within the brainstem, and the locus coeruleus, a major noradrenergic center regulating vigilance, stress responses, and attentional processes (Vandewalle et al., 2010). Functional neuroimaging studies further suggest interactions between light-sensitive pathways and large-scale neural networks involved in emotional appraisal and environmental monitoring, including components of the salience network. Modulation of these circuits may contribute to changes in perceived anxiety and physiological arousal (Kemp and Quintana, 2015). Second, autonomic responses to color may involve learned psychological associations. Across many cultures, blue is commonly associated with calmness, safety, stability, water, and open space, whereas red and yellow are often associated with alertness, danger, urgency, or increased activity (Elliot and Maier, 2014). Such associations may modulate emotional responses independently of purely physiological photoreceptor-mediated mechanisms (Pessoa, 2010). Third, cognitive appraisal may play an important role. In the present study, subjective evaluations demonstrated that most participants perceived blue light as relaxing and red or yellow light as stimulating. These perceptions may contribute to autonomic changes through top-down modulation of emotional processing and stress responses. Taken together, these observations suggest that both neurophysiological and psychological mechanisms may contribute to the effects of colored light exposure. The choice of a 10-min exposure duration was pragmatic and based on the operational constraints of the preoperative setting. Although significant autonomic and subjective changes were observed, the optimal duration of colored light exposure remains unknown. Longer exposures may produce different physiological effects, particularly given the time-dependent influence of light on non-visual neural pathways. Future studies should investigate dose-response relationships between exposure duration, light intensity, and emotional outcomes.

Although statistically significant changes in anxiety and HRV were observed, their clinical significance requires careful interpretation. The primary objective of this pilot study was exploratory rather than confirmatory. Consequently, the findings should not be interpreted as evidence that colored light alone can replace established approaches to perioperative anxiety management. Instead, they suggest that environmental lighting may represent a complementary strategy capable of modestly improving patient comfort and emotional regulation. Whether the observed autonomic changes translate into clinically meaningful outcomes remains unknown. Future studies should investigate potential effects on perioperative medication requirements, patient satisfaction, procedural tolerance, postoperative recovery, and healthcare resource utilization. Such outcomes may ultimately prove more relevant than isolated physiological measurements.

An important observation was the substantial variability in responses between participants.

Although blue light was associated with overall reductions in anxiety and increases in HRV, not all participants responded similarly. This variability likely reflects multiple factors including age, personality traits, prior experiences, cultural associations, emotional state, visual perception, and baseline autonomic function. The role of individual color preference deserves particular attention. Although individual preferences contributed modestly to subjective responses, the effects of lighting condition on anxiety and HRV remained significant after adjustment, suggesting that the observed physiological responses were not solely attributable to color preference. While adjustment for color preference did not materially alter the principal findings, subjective attitudes toward specific colors may still influence emotional responses. Future studies incorporating validated psychological instruments could provide a more comprehensive understanding of these relationships.

The choice of cataract patients presents both strengths and limitations. This population is highly relevant because cataract surgery is among the most frequently performed surgical procedures worldwide and is commonly associated with preoperative anxiety despite excellent outcomes. Furthermore, patients generally remain conscious throughout the perioperative process, making emotional regulation particularly important. However, age-related ocular changes may influence color perception. Cataract formation alters light transmission and spectral sensitivity, particularly in shorter wavelengths. Consequently, the physiological and psychological effects of colored light observed in this study may not be directly generalizable to younger populations or to patients without visual impairment.

Future investigations could compare responses before and after cataract extraction or across different age groups to better characterize these effects.

Several other limitations to the study must be acknowledged. Because participants were exposed sequentially to all lighting conditions, carryover and expectancy effects cannot be excluded. This within-subject exploratory design differs from a fully controlled between-subject design including a non-exposed control group. Because this study was exploratory and hypothesis-generating, no formal a priori power analysis was performed. Consequently, findings should be interpreted cautiously and confirmed in larger adequately powered trials. HRV was assessed using a consumer wearable device, which facilitated continuous non-invasive monitoring but may provide lower accuracy than standard ECG-derived HRV measurements. Furthermore, the specific device has not been formally validated in this perioperative setting. RMSSD primarily reflects parasympathetic modulation and does not provide a complete assessment of sympathetic nervous system activity. The absence of a direct sympathetic index therefore limits interpretation of autonomic balance.

The present pilot study should be viewed as a proof-of-concept investigation. Future randomized controlled trials should include larger and more diverse populations, validated ECG-based HRV monitoring, assessment of sympathetic nervous system activity, and comprehensive psychological profiling. Additional environmental variables such as light intensity, dynamic color transitions, natural daylight exposure, and architectural characteristics should also be explored. Ultimately, integrating physiological monitoring with environmental design may contribute to the development of evidence-based healing environments capable of improving both patient experience and clinical outcomes. Patient expectations, prior emotional associations with colors, and placebo/nocebo mechanisms may have influenced subjective anxiety ratings and were not specifically controlled in the present study. By allowing each participant to serve as their own control, the design minimized confounding related to baseline anxiety, autonomic tone, and subjective emotional responsiveness. However, sequential exposure to multiple lighting conditions may have introduced carryover effects, habituation phenomena, or expectancy bias. Because this pilot study included only 30 participants, a between-subject design would likely have been underpowered to detect modest physiological effects of colored light.

Future studies should consider parallel-group randomized controlled designs to confirm the present findings.

## Data Availability

The raw data supporting the conclusions of this article will be made available by the authors, without undue reservation.
